# Psychometric Properties of the Chinese Irrational Procrastination Scale: Factor Structure and Measurement Invariance Across Gender

**DOI:** 10.3389/fpsyg.2021.768581

**Published:** 2021-10-18

**Authors:** Amy Shaw, Jennifer J. Zhang

**Affiliations:** ^1^Department of Psychology, Faculty of Social Sciences, University of Macau, Taipa, Macau, China; ^2^Department of Psychology, University of Houston, Houston, TX, United States

**Keywords:** procrastination, irrational procrastination scale, validation study, confirmatory factor analysis, multigroup confirmatory factor analysis, measurement invariance, gender differences, Chinese college students

## Abstract

The present paper reports on the preliminary validation of a Chinese version of Steel’s Irrational Procrastination Scale (IPS). To this end, the nine items of the IPS were translated into Chinese and data were collected from a sample of 2,361 mainland Chinese college students. Confirmatory factor analysis (CFA) was used to examine the dimensional structure of the IPS, and multigroup CFA (MG CFA) was carried out to evaluate the measurement invariance across gender. Results revealed that the Chinese IPS had adequate internal consistency reliability, adhered to the one-factor structure, and exhibited strong or scalar invariance across the two gender subgroups, thereby providing support for the internal construct validity of the scale. Additionally, the IPS scores were found to be strongly and negatively related to the Conscientiousness personality trait while showing weak correlations with the other traits, which provided some support for the convergent and divergent validity of the Chinese IPS. Study limitations and future research directions (e.g., expanding the empirical evidence for the scale’s criterion-related validity) are discussed.

## Introduction

Procrastination, the voluntary delay of action despite knowing to become worse off or disadvantageous due to the delay (Steel, 2007, 2010), is viewed as an irrational behavior pattern often associated with negative outcomes such as lower task performance, decreased well-being, increased physical and mental suffering, and unnecessary feelings such as worry, discomfort, guilt, and stress (Tice and Baumeister, 1997; Steel, 2007, 2010; Klingsieck, 2013; Steel and Ferrari, 2013). As a prevalent issue among both the general public and student populations, procrastination has been frequently examined in epidemiological research *via* self-report measures. Such studies estimated that at least 20% of the general adult population experiences difficulties related to procrastination (Harriott and Ferrari, 1996) and 50–95% of the student population engages in procrastinatory behaviors on a daily basis (Day et al., 2000; Ellis and Knaus, 2002; Schouwenburg et al., 2004; Steel, 2007). According to Steel and Ferrari (2013), procrastinators are often aware of their own tendency to procrastinate while recognizing procrastination as negative and are also motivated to overcome or reduce it (Steel, 2007).

Given the prevalence and harmful consequences of procrastination, there has been a strong motive to develop and validate psychometrically sound measures for procrastination. Among a multitude of procrastination scales in the literature (see Steel, 2010, for an overview), the Irrational Procrastination Scale (IPS; Steel, 2010, 2002) was developed to focus on measuring one single unitary construct – the irrational or dysfunctional delay of intended behavior in the implemental phase (Steel, 2002, unpublished; Andreou, 2007) which was regarded as the most essential attribute of procrastination (Steel, 2007). The IPS contains nine items in total [e.g., “I delay tasks beyond what is reasonable” (Item 7)] all of which are intended to capture the construct of irrational procrastination. Due to its relatively short length, simple unidimensional structure, convenience to administer and score, and adequate psychometric properties (Steel, 2010; Steel and Ferrari, 2013), the IPS has not only become a commonly used and well-established scale for measuring procrastination in English-speaking countries but also attracted considerable attention from the international research community. Several recent adaptation and validation efforts have been made to investigate the psychometric properties of the IPS to support its use in other languages than English [e.g., Indonesian (Prayitno et al., 2013), French (Rebetez et al., 2014), Swedish (Rozental et al., 2014), Polish (Stępień and Topolewska, 2014), Norwegian (Svartdal, 2017), Spanish (Guilera et al., 2018), Russian (Klepikova and Kormacheva, 2019), and Korean (Kim et al., 2020)], but to our knowledge, no validation has been carried out in the Chinese context. Hence, the aim of the current study is to investigate the psychometric properties of the Chinese version of the IPS to provide support for its use in the Chinese settings which in turn would be helpful in facilitating procrastination research and prevention practices in China. Specifically, the internal consistency reliability and factor structure of the scale followed by its measurement invariance across gender as well as the relations to external variables are examined.

## Materials and Methods

### Participants and Procedure

This study was part of a larger project for investigating affect and performance. The current sample is comprised of *N*=2,361 mainland Chinese college students who took part in the study for extra credit. Participation was voluntary, anonymous, and confidential. After providing their written informed consent, participants completed a background information sheet that collected data concerning age and gender in addition to the translated and adapted Chinese version of the IPS and a personality measure, both of which will be elaborated upon in the following sections. Questionnaires were completed during class time and then collected on the spot. There were no missing data so that all participants were included in the final analysis sample (*N*=2,361). Their ages ranged from 18 to 22years with an average of 19.89 (*SD*=0.56). Among these participants, *N*=1,294 (54.8%) were male.

### The IPS-China Form

To construct the Chinese version of the nine-item IPS (Steel, 2010, 2002), the original English version of the IPS was first translated in to simplified Chinese by two independent bilingual translators whose mother tongue was Chinese (forward translation). The translators then discussed and resolved their discrepancies in the initial translation. Afterward, the interim Chinese IPS was retroverted into English by a native English speaker with excellent mastery of Chinese but without knowledge of the original English version (back translation). The back-translated English version was then compared to the original version to ensure that both versions shared the psychological meaning. Finally, all translated versions were discussed for clarification and consolidated in an expert review session, during which a few minor changes were made to the interim Chinese IPS. The items of the adapted Chinese IPS are presented in Table 1. We use the term “adapted” because the translation process emphasized the retainment of the original intent and essential meaning of the scale (Hambleton et al., 2005).

**Table 1:** Irrational procrastination scale (IPS) items in English and Chinese.

Item	English	Chinese (Simplified)
IPS1	I put things off so long that my well-being or efficiency unnecessarily suffers	我把事情拖得太久, 以至于我的幸福感或效率受到了不必要的负面影响
IPS2 (R)	If there is something I should do, I get to it before attending to lesser tasks	如果有我该做的事情, 我会先去做这件事, 然后再去处理其它次要的任务
IPS3	My life would be better if I did some activities or tasks earlier	如果我早点儿开始做某些活动或任务的话, 我的生活会更好
IPS4	When I should be doing one thing, I will do another	当我应该做某件事情时, 我反而会去做另一件事
IPS5	At the end of the day, I know I could have spent the time better	回顾当天, 我知道我本可以更好地使用我的时间
IPS6 (R)	I spend my time wisely	我明智地利用我的时间
IPS7	I delay tasks beyond what is reasonable	我拖延任务的时间超出了合理范围
IPS8	I procrastinate	我拖延
IPS9 (R)	I do everything when I believe it needs to be done	只要我认为某件事情需要完成, 我就会去做它

*Items designated with an (R) are reverse scored*.

As displayed in Table 1, following the format of the English version, the Chinese IPS contains nine items in total three of which are reversely scored (Items 2, 6, and 9). The participants also rated the IPS on a five-point Likert scale (1=Very seldom/Not true of me; 5=Very often true/True of me). In scoring, all items were coded such that higher numbered responses referred to “more” of the trait and the total score was computed by adding all responses to the nine items. Therefore, higher scores on the scale indicated greater levels of procrastination with a possible total score range of 9–45.

### The Personality Measure

To provide criterion-related validity evidence for the Chinese IPS adapted in the current study, we examined its relations to the Big-five personality traits (Costa and McCrae, 1992) which have been demonstrated to be relevant to procrastination in the literature. Specifically, two meta-analytic studies (Van Eerde, 2003; Steel, 2007) both found that procrastination strongly related to Conscientiousness, while exhibiting low or null relationships with the other traits. Validation studies for the IPS in other countries also revealed large and negative correlations between the IPS scores and Conscientiousness (Rebetez et al., 2014; Guilera et al., 2018). In this study, the Big-five personality traits were measured *via* the 50-item International Personality Item Pool scale (IPIP; Goldberg et al., 2006) which has been validated in the Chinese setting (Zheng et al., 2008). The IPIP items were administered with a five-point Likert scale (1=Very inaccurate description of me; 5=Very accurate description of me). All the trait subscales (with 10 items in each) had satisfactory estimated reliabilities (Cronbach’s alpha coefficients ranged from 0.81 to 0.91).

## Analyses and Results

### Descriptive Statistics

All planned analyses in the study were based on raw scores in R version 3.4.4 (R Core Team, 2018). The observed IPS total scores for this sample ranged between 9 and 45 with an average of 31.47 (*SD*=5.65, *N*=2,361), suggesting a slightly-greater-than-average level of irrational procrastination which accorded well with the general anticipation that college students tended to procrastinate more (Day et al., 2000; Steel, 2007). The skewness value was *S*=−0.29, and the kurtosis value was *K*=0.17 for the total score, both of which were well within the range of −1.00 to 1.00 to consider the score distribution as not deviating from normality (Tabachnick and Fidell, 2013). The distribution statistics suggested that the scale was sufficient in capturing different levels of irrational procrastination among college students and was not limited by any noticeable floor or ceiling effect. Not violating the normality assumption is also important to parameter estimation in the ensuing factor analysis (Kline, 2016).

### Reliability

Comparable to that of the original English version (*α*=0.91; Steel, 2010), the internal consistency reliability of the Chinese IPS (Cronbach’s *α*=0.91; McDonald’s *ω*=0.92) was excellent per the rule of thumb (Nunnally and Bernstein, 1994). As presented in Table 2, corrected item-total correlations of the nine items on the scale ranged from 0.55 to 0.75 and were all statistically significant at the 0.001 level, suggesting that all items functioned satisfactorily in terms of item discrimination (all correlations were >0.50 and in the expected direction; Nunnally and Bernstein, 1994; Clark and Watson, 1995). The high item-total correlations also indicated that all the nine items contributed well to the overall scale and appeared to be defining a central construct, providing some initial evidence for the unidimensionality of scale.

**Table 2:** Item-total correlations and factor loadings of the Chinese IPS.

Item	Standardized factor loading	Item-total correlation (corrected)
IPS1	0.80^*^	0.75^*^
IPS2 (R)	0.68^*^	0.63^*^
IPS3	0.58^*^	0.55^*^
IPS4	0.76^*^	0.72^*^
IPS5	0.79^*^	0.73^*^
IPS6 (R)	0.69^*^	0.65^*^
IPS7	0.65^*^	0.61^*^
IPS8	0.78^*^	0.73^*^
IPS9 (R)	0.67^*^	0.62^*^

* *denotes p<0.001*.

*Items designated with an (R) are reverse scored*.

### Factor Structure

The IPS was originally theorized and developed as a unidimensional scale (Steel, 2010, 2002), and subsequent empirical examinations of different language versions of the IPS have largely pointed to a single-factor structure of the scale (e.g., Svartdal et al., 2016; Svartdal, 2017; Kim et al., 2020; see also Prayitno et al., 2013; Rozental et al., 2014). Therefore, we expected the Chinese IPS to be also unidimensional and therefore, conducted a confirmatory factor analysis (CFA) using the *lavaan* package version 0.6–2 (Rosseel, 2018) in R as a confirmatory test of the unidimensionality. Accordingly, we fit a single-factor model in which all of the nine items loaded on one factor with the robust weighted least squares mean and variance-adjusted (WLSMV) estimator, given the ordinal categorical nature of the response data (Muthén et al., 1997, unpublished; Finney and DiStefano, 2006). Criteria to determine the overall model fit included the Chi-square statistic (*χ*^2^) and the following goodness-of-fit indices: the comparative fit index (CFI; Bentler, 1990), the Tucker-Lewis index (TLI; Tucker and Lewis, 1973), the root-mean-square error of approximation (RMSEA; Steiger, 1990), and the standardized root-mean-square residual (SRMR). Because of the large sample size in the current study, it would be inappropriate to use Chi-square significance testing for model fit determination as even a small difference would be found to be statistically significant within large samples (Brown, 2015). We thus primarily relied on the inspection of the goodness-of-fit indices employing the standards recommended in the literature; conventionally, CFI≥0.95, TLI≥0.95, RMSEA≤0.06, and SRMR≤0.08 would indicate a very good fit of the model to the data (Browne and Cudeck, 1993; Hu and Bentler, 1999; Kline, 2016). All the goodness-of-fit indices in this study were excellent [*χ*^2^(27)=260.16, *p*<0.001, CFI=0.990, TLI=0.988, SRMR=0.048, and RMSEA=0.060], demonstrating that the parsimonious single-factor model represented the data satisfactorily. Moreover, as shown in Table 2, all items exhibited good factor loadings on the latent factor (ranging from 0.58 to 0.80 and statistically significant at the 0.001 level), further supporting the adequate discriminating power of the items across the latent range of the construct. Also, there was no modification index suggested for any type of cross-loading, and thus, each item was considered as the exclusive indictor of the intended latent variable (i.e., irrational procrastination).

### Measurement Invariance Across Gender

Once the unidimensionality was confirmed, we proceeded to test the one-factor model on male and female students separately to evaluate the measurement equivalence or invariance across gender subgroups of the Chinese IPS. Although no gender difference was observed in the IPS scores in this sample [IPS-Total-Male=31.48 vs. IPS-Total-Female=31.45, *t*(2359)=0.13, *p*=0.89], previous research (e.g., Steel and Ferrari, 2013; Beutel et al., 2016; Guilera et al., 2018) has identified gender differences in procrastination with males procrastinating more than females (albeit the effect sizes were small). Conclusions about gender differences (or lack thereof) under study cannot be settled until measurement invariance (a prerequisite for meaningful score comparisons across groups) of the instrument has been established (Millsap and Olivera-Aguilar, 2012; Brown, 2015; Kline, 2016).

A multigroup men vs. women CFA (MG CFA) was thus performed over gender subgroups using standard procedures to test for configural invariance (equal form), metric/weak invariance (equal loadings), and scalar/strong invariance (equal intercepts) in increasingly restrictive steps (Millsap and Olivera-Aguilar, 2012; Kline, 2016). Strict invariance (equal residual errors) was not tested because it is often considered as overly stringent and unnecessary by methodologists and has rarely been observed in practice (e.g., Cheung and Lau, 2012; Brown, 2015; Van De Schoot et al., 2015). In determining invariance, we placed more emphasis on changes in model fit indices (the CFI, SRMR, and RMSEA values; Cheung and Rensvold, 2002) relative to the Chi-square difference significance tests of the nested models between each successive step, because one would expect the likelihood ratio-based Chi-square tests to be significant (an indication of non-invariance) with a large sample size (Brown, 2015). To determine that measurement invariance holds for the more constrained model in each comparison, ΔCFI should be less than 0.010, ΔSRMR should be less than 0.01, and ΔRMSEA should not exceed 0.015 (Cheung and Rensvold, 2002; Chen, 2007). Table 3 summarizes the MG CFA results. Overall, the fit statistics supported the measurement invariance for the Chinese IPS across gender in metric invariance (ΔCFI=−0.002, ΔSRMR=0.003, and ΔRMSEA=0.001) and scalar invariance (ΔCFI=−0.001, ΔSRMR=0.003, and ΔRMSEA=−0.002) as the changes in the fit indices did not indicate meaningful decrement in model fit (Cheung and Rensvold, 2002; Chen, 2007). Therefore, we decided that the Chinese IPS assessed irrational procrastination equivalently across gender, which ensured meaningful score comparisons between men and women.

**Table 3:** Measurement invariance across gender through multigroup confirmatory factor analysis (MG CFA).

Models and comparisons	Fit statistics							
	*χ*^2^(*df*)	CFI	SRMR	RMSEA	Δ*χ*^2^(Δ*df*)	ΔCFI	ΔSRMR	ΔRMSEA
Configural model (Model 1)	347.92 (54)^*^	0.984	0.053	0.067				
Metric model (Model 2)	407.45 (62)^*^	0.982	0.056	0.068				
Scalar model (Model 3)	442.91 (70)^*^	0.981	0.059	0.066				
Configural vs. Metric: Model 2 – Model 1					59.53 (8)^*^	−0.002	0.003	0.001
Metric vs. Scalar: Model 3 – Model 2					35.46 (8)^*^	−0.001	0.003	−0.002

*CFI = comparative fit index; SRMR = standardized root-mean-square residual; and RMSEA = root-mean-square error of approximation*.

* *denotes p<0.001*.

### Relations to Personality Traits

By examining Pearson’s correlation coefficients between the IPS scores and external construct-related variables (i.e., the Big-five personality traits), we evaluated the concurrent validity of the Chinese IPS. In line with past works on the personality correlates of the IPS total scores (e.g., Van Eerde, 2003; Steel, 2007; Rebetez et al., 2014; Guilera et al., 2018), procrastination measured by the Chinese IPS was found to be negatively and strongly related to Conscientiousness (*r*=−0.61, *p*<0.001) while showing weak relations to the other traits: Extraversion (*r*=−0.13, *p*<0.001), Agreeableness (*r*=−0.12, *p*<0.001), Emotional Stability (*r*=−0.20, *p*<0.001), and Openness to Experience (*r*=0.11, *p*<0.001). Therefore, the Chinese IPS scores were correlated with the Big-five personality traits in the expected direction and magnitudes according to the standards suggested by Cohen (1988).

## Discussion

The study purpose was to investigate the psychometric properties of the IPS, one of the most widely used procrastination assessments in the current literature, to provide validity evidence for its use in the Chinese context. In line with the aim of the study, we first translated and adapted the original IPS into a Chinese version and then evaluated the internal consistency reliability, factor structure, and gender-based measurement invariance of the scale. Overall, the results suggested promising psychometric properties of the Chinese version of the IPS. In agreement with previous research (e.g., Steel, 2010; Rozental et al., 2014; Svartdal et al., 2016; Svartdal and Steel, 2017; Guilera et al., 2018), we found adequate internal consistency reliability of the nine-item scale within this sample. Regarding the dimensionality, the CFA results confirmed the one-factor structure of the IPS, which fit well with the original idea of the scale (as a unidimensional measure of irrational procrastination) put forward by Steel (2010, 2002) as well as conclusions from prior validation studies in other languages and countries/regions (e.g., Svartdal et al., 2016; Svartdal, 2017; Kim et al., 2020; Shaw and Zhang, 2021). Furthermore, measurement invariance held across gender, demonstrating that the Chinese IPS was not biased against men or women and that any gender difference in the scale scores would be attributable to the underlying psychological construct itself. As such, the study also provided evidence for the appropriate use of the Chinese IPS as an instrument of measuring gender differences in irrational procrastination. In addition, the observed correlates with the Big-five personality traits were as expected and consistent with the findings reported in recent works (Van Eerde, 2003; Steel, 2007; Rebetez et al., 2014; Guilera et al., 2018), providing some evidence for the scale’s convergent and divergent validity.

Several study limitations should be taken into account while reviewing the present findings. First, the Chinese IPS clearly needs further examination and validation, especially with respect to the evaluation of criterion-related validity. Future investigations of its relations with external variables such as other currently available Chinese procrastination instruments [e.g., the Aitken Procrastination Inventory originally developed by Aitken (1982, unpublished) and recently adapted into Chinese by Chen et al. (2008), the Tuckman Procrastination Scale originally developed by Tuckman (1991) and then validated in the Chinese setting by Zhang and Zhang (2007)] would be highly desirable for expanding its convergent validity. Future research may also assess the predictive validity of the Chinese IPS on outcome measures such as subjective well-being measured by the Satisfaction With Life Scale (SWLS; Diener et al., 1985; Chinese version validated by Bai et al., 2011), an outcome variable that has been well-demonstrated to be negatively affected by procrastination (Steel, 2010; Sirois and Tosti, 2012; Rebetez et al., 2014; Beutel et al., 2016; Svartdal, 2017; Guilera et al., 2018). Second, to complement the present study utilizing the classical test theory (CTT) and factor analysis, future research could apply the more modern item response theory (IRT; Embretson and Reise, 2000) techniques for additional item-level diagnostic metrics (e.g., Shaw et al., 2021; see also Shaw et al., 2020 for a recent discussion on applying advanced IRT models in the validation and refinement of measures). Third, because we adopted a college student sample, it is unclear whether the patterns of the present findings may generalize to other populations such as working adults. Given that past works have found the student population to be particularly prone to procrastination (Day et al., 2000; Steel, 2007; Steel and Ferrari, 2013; Hicks and Storey, 2015; Svartdal, 2017), further research with more heterogeneous samples is warranted to try to replicate the current findings. Fourth, even though considerable effort was devoted to the translation and adaption process in order to retain the core meaning of the original scale and to ensure the content quality of the Chinese IPS items, it is likely that linguistic or cultural differences still introduced construct-irrelevant biases to the process which in turn, affected respondents’ interpretation of the items. To help clarify such potential issues, future studies may conduct cognitive interviews which presumably could provide direct and valuable insights into how the target construct (i.e., irrational procrastination) might be regarded by participants in their individual response processes (Krosnick, 1999; Hambleton et al., 2005; Shaw et al., 2020).

In sum, as an initial validation study, our paper presented preliminary validity evidence supporting the use of the IPS in the Chinese context. The findings are in accordance with conclusions from studies that corroborated the psychometric soundness of the IPS in other countries (e.g., Svartdal et al., 2016; Svartdal, 2017; Guilera et al., 2018; Kim et al., 2020; Shaw and Zhang, 2021). At present, there has been accumulated evidence globally that suggests the utility of the IPS as a brief and valid scale for assessing procrastination. In response to the growing need of useful and easy-to-administer procrastination scales in an international context, we encourage further investigations of the Chinese IPS to strengthen and expand its validity evidence as well as call for more attempts at adapting and validating the IPS in additional non-English speaking populations. Collectively these validations studies may allow for future cross-cultural comparisons on procrastination with a well-established instrument.

## Data Availability Statement

The original contributions presented in the study are included in the article, further inquiries can be directed to the corresponding author.

## Author Contributions

AS and JZ contributed to the conceptualization, data collection, data analysis, and manuscript preparation.

## Conflict of Interest

The authors declare that the research was conducted in the absence of any commercial or financial relationships that could be construed as a potential conflict of interest.

## Publisher’s Note

All claims expressed in this article are solely those of the authors and do not necessarily represent those of their affiliated organizations, or those of the publisher, the editors and the reviewers. Any product that may be evaluated in this article, or claim that may be made by its manufacturer, is not guaranteed or endorsed by the publisher.
